# An Analysis of Pregnancy-Related Mortality in the KEMRI/CDC Health and Demographic Surveillance System in Western Kenya

**DOI:** 10.1371/journal.pone.0068733

**Published:** 2013-07-16

**Authors:** Meghna Desai, Penelope A. Phillips-Howard, Frank O. Odhiambo, Abraham Katana, Peter Ouma, Mary J. Hamel, Jackton Omoto, Sheila Macharia, Annemieke van Eijk, Sheila Ogwang, Laurence Slutsker, Kayla F. Laserson

**Affiliations:** 1 Malaria Branch, KEMRI/CDC Research and Public Health Collaboration, Kisumu, Kenya; 2 Center for Global Health, Centers for Disease Control and Prevention, Atlanta, GA, United States of America; 3 Department of Clinical Sciences, Liverpool School of Tropical Medicine, Liverpool, United Kingdom; 4 Health and Demographic Surveillance System Branch, KEMRI/CDC Research and Public Health Collaboration, Kisumu, Kenya; 5 Department of Obstetrics and Gynaecology, Siaya District Hospital, Ministry of Health, Siaya, Kenya; 6 Office of Population and Health, US Agency for International Development, Nairobi, Kenya; Tulane University School of Public Health and Tropical Medicine, United States of America

## Abstract

**Background:**

Pregnancy-related (PR) deaths are often a result of direct obstetric complications occurring at childbirth.

**Methods and Findings:**

To estimate the burden of and characterize risk factors for PR mortality, we evaluated deaths that occurred between 2003 and 2008 among women of childbearing age (15 to 49 years) using Health and Demographic Surveillance System data in rural western Kenya. WHO ICD definition of PR mortality was used: “the death of a woman while pregnant or within 42 days of termination of pregnancy, irrespective of the cause of death”. In addition, symptoms and events at the time of death were examined using the WHO verbal autopsy methodology. Deaths were categorized as either (i) directly PR: main cause of death was ascribed as obstetric, or (ii) indirectly PR: main cause of death was non-obstetric. Of 3,223 deaths in women 15 to 49 years, 249 (7.7%) were PR. One-third (34%) of these were due to direct obstetric causes, predominantly postpartum hemorrhage, abortion complications and puerperal sepsis. Two-thirds were indirect; three-quarters were attributable to human immunodeficiency virus (HIV/AIDS), malaria and tuberculosis. Significantly more women who died in lower socio-economic groups sought care from traditional birth attendants (p = 0.034), while less impoverished women were more likely to seek hospital care (p = 0.001). The PR mortality ratio over the six years was 740 (95% CI 651–838) per 100,000 live births, with no evidence of reduction over time (χ^2^ linear trend = 1.07; p = 0.3).

**Conclusions:**

These data supplement current scanty information on the relationship between infectious diseases and poor maternal outcomes in Africa. They indicate low uptake of maternal health interventions in women dying during pregnancy and postpartum, suggesting improved access to and increased uptake of skilled obstetric care, as well as preventive measures against HIV/AIDS, malaria and tuberculosis among all women of childbearing age may help to reduce pregnancy-related mortality.

## Introduction

Improving maternal health is a high priority for the United Nations international development agenda. As part of the fifth Millennium Development Goal (MDG5) set in 2000, maternal mortality is targeted for substantial reduction by 2015 [1]. Unfortunately, progress in sub-Saharan Africa (SSA) towards this target has stalled. In 2005, the majority of countries in the region were estimated to have maternal mortality ratios over 300 maternal deaths per 100,000 live births [2]. Kenya exemplifies this lack of progress in recent years, with an estimated maternal mortality ratio of 488 maternal deaths per 100,000 live births reported for 2008–2009 [3] compared to 414 deaths per 100,000 in 2003 [4]. These estimates translate to approximately 7,700 maternal deaths annually in Kenya.

The leading causes of maternal mortality in SSA are obstetric complications such as severe bleeding, obstructed labor, infection, and hypertensive disorders of pregnancy [5]. Major contributors also include indirect causes such as HIV/AIDS, malaria and anemia [5], [6]. Unsafe abortion, though poorly documented, is also a major factor which may account for up to 14% of all maternal deaths worldwide [7]. Many SSA countries would benefit from having population based maternal mortality data to prioritize and better focus interventions to reduce maternal mortality.

Kenya, like many other developing countries, suffers from an incomplete death registration system and inaccuracy in the ascertainment of the causes of death, including maternal deaths. Although studies on maternal mortality have been conducted in informal settlements [8], there are no published reports of the causes of pregnancy-related or maternal deaths from rural Kenya. We utilized verbal autopsy (VA) data collected in the Health and Demographic Surveillance System (HDSS) site in Nyanza Province, rural western Kenya to estimate the burden of and characterize risk factors for pregnancy-related mortality. We also examined care reportedly accessed by women with a pregnancy-related death and compared it, where available or appropriate, with women attributed a non-pregnancy-related death.

## Materials and Methods

### Study Site and Population

The study site is located in a rural part of Nyanza Province in western Kenya in the areas of Asembo (Rarieda District), Karemo (Siaya District) and Gem (Gem District) in Siaya County [9], [10]. The population comprises approximately 225,000 individuals living in 385 villages spread over 700 km^2^. It has a typical rural African population age distribution with 44.6% under 15 years of age, and only 5.5% over 65 years of age. By 2008, a total of 94,106 persons were aged 15–49 years, 41.7% of the population, of whom 50,820 (54%) were women of childbearing age. The population is culturally homogeneous; over 95% are members of the Luo ethnic community and live through subsistence farming and local trading. The society is polygynous, with males frequently having more than one wife, each of whom lives in a separate house with young children within a single compound. HDSS residents (defined as those residing in the study area for at least 4 consecutive months or infants born to residents) are visited every four months. Previous studies identified the population to be generally very poor [11].

Malaria is endemic in this area, and transmission occurs throughout the year. The prevalence of malaria among individuals over 15 years of age ranged between 10–20% in the period 2006 to 2008 (KEMRI/CDC, unpublished observations). HIV, tuberculosis (TB) and geohelminth prevalence are also some of the highest in the country. In the period between 2003 and 2008, in the HDSS, the prevalence of HIV among girls between 15–19 years of age was estimated at 8.6% [12], the prevalence of TB in individuals over 15 years of age was 600/100,000 [13], and geohelminth prevalence in pregnant women was recorded to be as high as 76.2% [14]. During this time period, HIV treatment and care centers expanded [15], the coverage of malaria interventions (insecticide-treated bednets and intermittent preventive treatment in pregnancy) increased [16], and training of healthcare workers to provide focused antenatal care was rolled out [17]. There was a gradual shift in Kenyan policy from allowing traditional birth attendants (TBAs) to conduct deliveries to redefining their role as referral agents and birth companions. There are 36 health facilities in the HDSS, including one district hospital, two privately owned hospitals, 11 health centers and 22 dispensaries.

### Health and Demographic Surveillance System (HDSS)

The entire population is registered and geo-spatially located within the HDSS [10]. A household census (“round”) is conducted three times per year to capture pregnancies, births, deaths, and internal migration. Socio-economic status (SES), educational and marriage status data are collected every two years from all HDSS residents. Demographic data are used to provide mid-year denominators per 5-year age group, stratified by gender and study area. Deaths are captured in two ways. First, village reporters report all deaths to HDSS field supervisors as they occur. Second, community interviewers record any deaths that occurred during the prior 4 months at each routine HDSS round. Field staff then visit the GPS-located coded households at least one month after the reported death to validate deaths and record events surrounding death using VA.

### Verbal Autopsy (VA)

VAs [18] are administered to the primary caregiver of the deceased. A standardized questionnaire is used [19], to cover demographic and personal history, pre-mortem illness signs and symptoms, and events surrounding the death. VA is conducted for all deaths. The adult questionnaire is restricted to persons aged 15 years and above. For this analysis, we included women of childbearing ages: 15 to 49 years. Deaths were linked to HDSS data including socio-demographic, educational, marital status, and occupational information. For all deaths, VA information was reviewed independently and conflicts resolved by at least two clinical officers (equivalent to physician assistants in the U.S.A.) and one underlying cause of death assigned. Further details of the VA methodology used in the HDSS have been provided elsewhere [20]. Through the year 2007, VA questionnaires asked for information on miscarriage related to both spontaneous and induced abortions. In 2008, the standardized WHO VA questionnaire which only asked women to report induced abortions was adopted. However, as abortion is illegal in Kenya, we assume that the data gathered in 2008 predominantly capture spontaneous miscarriage. VA data are limited in their ability to differentiate between miscarriage and abortions, thus we do not present data separately by these categories.

### Ethical Considerations

Following cultural customs, compound heads provide written consent for all compound members to participate in the HDSS activities. Any individual can refuse to participate at any time. The HDSS protocol and consent procedures, including surveillance and VA, are approved by KEMRI and CDC Institutional Review Boards annually.

### Data Handling and Analyses

Data analyzed included all deaths in the HDSS occurring between January 1, 2003 and December 31, 2008 among female residents aged 15–49 years at the time of death. Karemo area (Siaya County), the immediate catchment area of the Siaya District Hospital, was included in the HDSS in 2008 only. The following WHO ICD definition of pregnancy-related (PR) mortality was used: “*the death of a woman while pregnant or within 42 days of termination of pregnancy, irrespective of the cause of death*”. Deaths were further categorized as either (i) directly PR, where the main cause of death determined by VA was obstetric, or (ii) indirectly PR, where the main cause of death ascribed through VA included any non-obstetric cause: infectious, non-infectious, or external causes.

Data analyses were conducted using SPSS for Windows (Release v18.0), and EpiInfo Stat Calc (CDC Atlanta, USA). In the absence of comparative data among survivors, within-death comparisons were made between PR and non-PR deaths to explore differences in characteristics, subdividing analyses into the WHO grouping of died in pregnancy, died after miscarriage/abortion, and died within 42 days of pregnancy. Key social and demographic characteristics included marital status (ever married; divorced or widowed at time of death), education (attended and completed primary school; attended secondary school), SES, and place of death (home, health facility, hospital, on route to/from hospital/health facility). A hospital is a district level or above facility, and a health facility is a local lower level facility. Routinely collected SES indicators such as occupation of household head, primary source of drinking water, use of cooking fuel, in-house assets (e.g. lantern lamp, sofa, bicycle, radio and television) and livestock (poultry, pigs, donkey cattle, sheep and goats) were used to calculate a wealth index as a weighted average using multiple correspondence analysis [21]. This was used to rank households into wealth quintiles with the first quintile representing the poorest and the fifth representing the least poor; for some analyses we collapsed into most (quintiles 1–2) and least (quintiles 3–5) poor. The significance of changes in rates over time was examined using Mantel Haenszel χ^2^ for linear trend. Differences between groups were determined using Pearson’s χ^2^ test and Fisher’s Exact test for small numbers, and a p-value of <0.05 was considered statistically significant. The pregnancy-related mortality ratio (PRMR) was calculated as the number of deaths among women of childbearing years (15–49 years) over the total number of live births to women of the same age range per year.

The HDSS data are stored securely and, through a formal process of data sharing established at KEMRI/CDC, are available for access to the scientific public two years after the data are cleaned and frozen.

## Results

### Comparison of PR and Non-PR Deaths

Between January 1, 2003 and December 31, 2008, 3,223 women aged 15–49 years died in the HDSS; 249 (8%) were classified as PR deaths. Among all PR deaths, 92 (37%) occurred during pregnancy, 54 (22%) following a miscarriage, 37 (15%) in the immediate postpartum period (defined as within 24 hours of delivery), and 66 (27%) between 1 and 42 days postpartum. Over half of the PR deaths occurred in women aged 20–29 years, compared to approximately one-third of non-pregnancy-related (non-PR) deaths in this age category (Table 1). Almost all (96%) of PR deaths occurred in women who attended primary school, with 63% completing primary; the proportions who attended or completed primary education were significantly greater in PR deaths compared with non-PR deaths (Table 1). Although only 20% of women dying from PR causes attended any secondary education, this was significantly higher than non-PR deaths (20% vs. 14%, p = 0.004). Small non-significant variations in PR deaths occurred by year in comparison to the significant decline in the number of non-PR deaths observed over the same time period (Table 1). Marriage at the time of death was significantly higher among PR compared with non-PR deaths (66% vs. 45%, p<0.001). Of note, 61% of PR deaths occurred at home and 34% at a health facility or hospital, compared with proportions of 83% and 15%, respectively, among non-PR deaths. Among PR deaths, a further 6% died on the journey to or from a health facility. Between 2003 and 2008, there was a trend towards fewer PR and non-PR deaths occurring in the home (PR deaths: 68% to 46%, p = 0.007; non-PR deaths: 89% to 76%, p<0.001). Lower SES was significantly associated with a pregnancy-related death occurring at home (66% in low SES versus 57% in high SES; p = 0.021).

**Table 1:** Distribution of pregnancy-related and non-pregnancy-related deaths of women 15–49 years by socio-demographic and health-related characteristics.^a^

		Pregnancy-Related Deaths				All Pregnancy-Related Deaths	All Non- Pregnancy-Related Deaths	?^2^	p value
				Post-partum					
		DuringPregnancy	AfterMiscarriage/Abortions^b^	<24 hrs	1–42 days				
		n = 92	n = 54	n = 37	n = 66	N = 249	N = 2974		
Age at death	15–19	7 (7.6)	1 (1.9)	6 (16.2)	13 (19.7)	27 (10.8)	136 (5.1)	18.27	<0.001
	20–24	25 (27.2)	10 (18.5)	8 (21.6)	19 (28.8)	62 (24.9)	415 (14.8)		
	25–29	23 (25.0)	22 (40.7)	8 (21.6)	15 (22.7)	68 (27.3)	634 (21.8)		
	30–34	20 (21.7)	10 (18.5)	3 (8.1)	12 (18.2)	45 (18.1)	532 (17.9)		
	35–39	9 (9.8)	6 (11.1)	7 (18.9)	6 (9.1)	28 (11.2)	438 (14.5)		
	40–44	8 (8.7)	4 (7.4)	4 (10.8)	1 (1.5)	17 (6.8)	435 (14.0)		
	45–49	0 (0.0)	1 (1.9)	1 (2.7)	0 (0.0)	2 (0.8)	384 (12.0)		
SES (MCA)^c^	(poorest)1	21 (23.9)	11 (22.4)	7 (22.6)	17 (27.0)	56 (24.2)	600 (21.6)	5.13	0.274
	2	16 (18.2)	8 (16.3)	5 (16.1)	14 (22.2)	43 (18.6)	583 (21.0)		
	3	20 (22.7)	11 (22.4)	7 (22.6)	8 (12.7)	46 (19.9)	595 (21.4)		
	4	13 (14.8)	7 (14.3)	5 (16.1)	12 (19.0)	37 (16.0)	532 (19.2)		
	5	18 (20.5)	12 (24.5)	7 (22.6)	12 (19.0)	49 (21.2)	464 (16.7)		
Area of residence	Asembo	34 (37.0)	19 (35.2)	12 (32.4)	25 (37.9)	90 (36.1)	1175 (39.5)	1.11	0.574
	Gem	53 (57.6)	35 (64.8)	19 (51.4)	36 (54.5)	143 (57.4)	1624 (54.6)		
	Karemo^d^	5 (5.4)	0 (0.0)	6 (16.2)	5 (7.6)	16 (6.4)	175 (5.9)		
Attend Primary	Yes	61 (95.3)	36 (100)	26 (92.9)	44 (95.7)	167 (96.0)	1406 (82.9)	20.20	<0.001
	No	3 (4.7)	0	2 (7.1)	2 (4.3)	7 (4.0)	290 (17.1)		
Complete Primary	Yes	40 (62.5)	25 (69.4)	17 (60.7)	27 (60.0)	109 (62.6)	709 (41.8)	27.85	<0.001
	No	24 (37.5)	11 (30.6)	11 (39.3)	19 (40.0)	65 (37.4)	987 (58.2)		
Attend Secondary	Yes	19 (21.3)	16 (30.8)	4 (11.4)	9 (14.8)	48(20.3)	329(13.5)	8.18	0.004
	No	70 (78.7)	36 (69.2)	31 (88.6)	52 (85.2)	189 (79.7)	2111 (86.5)		
Year of death	2003	13 (14.1)	19 (35.2)	5 (13.5)	7 (10.6)	44 (17.7)	568 (19.1)	14.77	0.011
	2004	16 (17.4)	12 (22.2)	2 (5.4)	12 (18.2)	42 (16.9)	557 (18.7)		
	2005	9 (9.8)	5 (9.3)	6 (16.2)	6 (9.1)	26 (10.4)	512 (17.2)		
	2006	25 (27.2)	10 (18.5)	6 (16.2)	11 (16.7)	52 (20.9)	440 (14.8)		
	2007	11 (12.0)	4 (7.4)	5 (13.5)	11 (16.7)	31 (12.4)	375 (12.6)		
	2008	18 (19.6)	4 (7.4)	13 (35.1)	19 (28.8)	54 (21.7)	522 (17.6)		
Married at death	Yes	60 (65.2)	29 (53.7)	31 (83.8)	43 (65.2)	163 (65.5)	1149 (44.5)	40.06	<0.001
	No	32 (34.8)	25 (46.3)	6 (16.2)	23 (34.8)	86 (34.5)	1432 (55.5)		
Place died	Home	56 (60.9)	37 (68.5)	20 (54.1)	38 (57.6)	151 (60.6)	2149 (83.0)	88.24	<0.001
	To/from HHF	7 (7.6)	0 (0.0)	3 (8.1)	4 (6.1)	14 (5.6)	38 (1.5)		
	HF	12 (13.0)	2 (3.7)	6 (16.2)	9 (13.6)	29 (11.6)	146 (5.6)		
	Hospital	17 (18.5)	15 (27.8)	8 (21.6)	15 (22.7)	55 (22.1)	235 (9.1)		
	Other	0 (0.0)	0 (0.0)	0 (0.0)	0 (0.0)	0 (0.0)	20 (0.8)		
Distance to HF^e^	0–1000 m	19 (20.7)	6 (11.1)	3 (8.1)	14 (21.2)	42 (16.9)	601(20.2)	3.97	0.41
	1001–2000 m	37 (40.2)	14 (25.9)	14 (37.8)	22 (33.3)	87 (34.9)	945(31.8)		
	2001–3000 m	26 (28.3)	21 (38.9)	13 (35.1)	22 (33.3)	82 (32.9)	1051(35.3)		
	3001–4000 m	10 (10.9)	13 (24.1)	7 (18.9)	5 (7.6)	35 (14.1)	343 (11.5)		
	4001–5000 m	0 (0)	0 (0)	0 (0)	3 (4.5)	3 (1.2)	25 (0.8)		
HIV/AIDS as cause		23 (25.0)	28 (51.9)	2 (5.4)	21 (31.8)	74 (29.7)	1382 (46.5)	46.6	<0.001
Malaria as cause		14 (15.2)	3 (5.6)	0 (0)	5 (7.6)	22 (8.8)	178 (6.0)	15.8	0.003
TB as a cause		9 (9.8)	2 (3.7)	1 (2.7)	4 (6.1)	16 (6.4)	458 (15.5)	16.3	0.003

a Data provided are complete for some variables (gender, age, year death), but for others are not always completed on VA form.

b Prior to 2008, VA questionnaire asked for all miscarriages (spontaneous or induced). As of 2008, the WHO VA questionnaire was adopted which only asked for induced abortions.

c SES (MCA) - socio-economic status (multiple correspondence analysis, in quintiles, 1 = poorest; 5 = least poor).

d Only generated in 2008.

e Distance to nearest health facility.

### Estimation of Pregnancy-related Mortality

**Figure 1:**
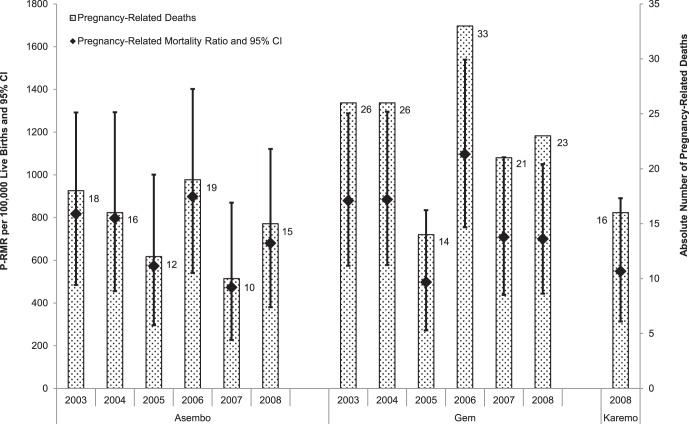
Pregnancy-related mortality ratio in women 15–49 years by year of death and area.

**Figure 2:**
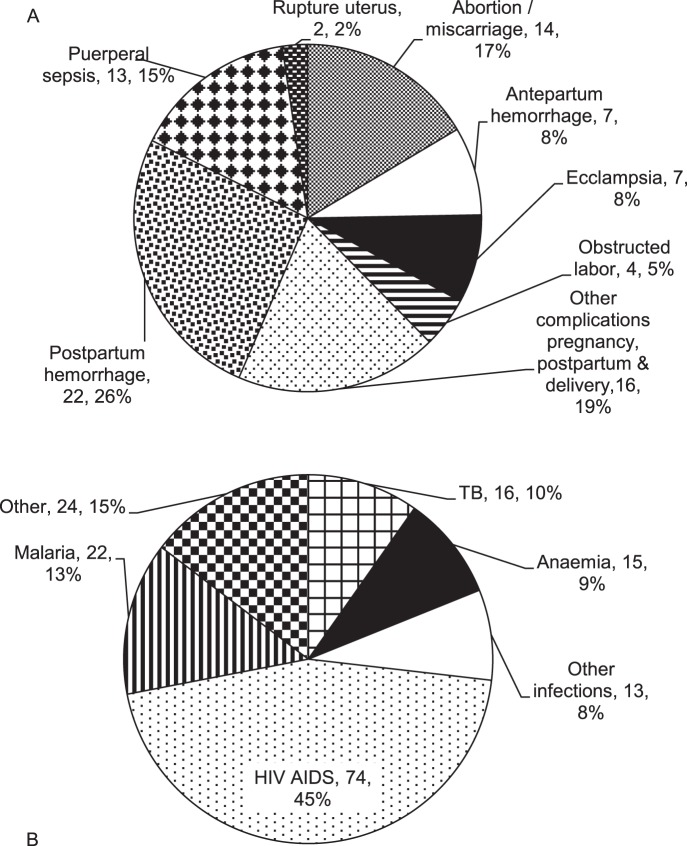
Classification of major causes of pregnancy-related deaths. A. Directly Ascribed Pregnancy-Related Mortality Causes B. Indirectly Ascribed Pregnancy-Related Mortality Causes.

Figure 1 presents estimates for the PRMR over time and study area. The total number of live births recorded in the HDSS between 2003 and 2008 was 34,103, yielding an estimated PRMR of 740 (95% CI 651–838) per 100,000 live births during the six year period. The lowest PRMR was in 2005 (524) and the highest in 2006 (1003) with no overall secular trend (χ^2^ linear trend = 1.07; p = 0.3). About one-third (34%) of all PR deaths were due to directly ascribed (i.e. obstetric) causes, and two-thirds (66%) to indirect or non-obstetric causes (Figure 2). The obstetric PRMR was 253 (95% CI 202–313) per 100,000 live births.

### Causes of Pregnancy-related Death by Verbal Autopsy

The proportion of PR deaths due to specific direct causes varied from 31% to 46% during the time period; these changes were not statistically significant (p = 0.45). The leading causes of direct PR deaths were postpartum hemorrhage (PPH, 26%), complications from abortion/miscarriage (17%), and puerperal sepsis (15%) (Figure 2a). A further 19% of the directly ascribed pregnancy-related mortality causes was constituted by other complications. Over one-third (34%) of the PR deaths from a direct maternal cause occurred within 24 hours of delivery (Table 2). Over 40% of all PR deaths due to a miscarriage or an induced abortion were in women aged 25–29 years.

**Table 2:** Distribution of direct causes of death by phase of pregnancy.

	Died inPregnancy	Died aftermiscarriage/abortion	Died within 24hours after delivery	Died 1–42 daysafter delivery	Total
Abortion/miscarriage	3 (21.4)	10 (71.4)	0 (0.0)	1 (7.1)	14
Antepartum hemorrhage	5 (71.4)	0 (0.0)	1 (14.3)	1 (14.3)	7
Ecclampsia	7 (100)	0 (0.0)	0 (0.0)	0 (0.0)	7
Obstructed labor	1 (16.7)		3 (50.0)	2 (33.3)	6
Other complications pregnancy,post-partum & delivery	6 (37.5)	3 (18.8)	4 (25.0)	3 (18.8)	16
Postpartum hemorrhage	0 (0.0)	0 (0.0)	20 (90.9)	2 (9.1)	22
Puerperal sepsis	0 (0.0)	1 (7.7)	1 (7.7)	11 (84.6)	13
Total	22 (25.9)	14 (16.5)	20 (23.5)	20 (23.5)	85

Among all pregnancy-related deaths, HIV/AIDS, malaria, and TB contributed 30%, 9%, and 6% of deaths, respectively (Table 1). Among indirect PR deaths, 45% were ascribed to HIV/AIDS, 13% to malaria, and 10% to TB (Figure 2b). Of note, PR deaths ascribed to HIV/AIDS as the direct cause occurred among significantly younger women (mean 29.6 years, SD 5.1) compared with HIV/AIDS ascribed non-PR deaths (mean age 34.3 years, SD8.3). The majority of indirect PR deaths (43%) occurred during pregnancy and nine deaths had an undetermined cause (Table 3).

**Table 3:** Distribution of indirect causes of death by phase of pregnancy.

	Died inPregnancy	Died aftermiscarriage/abortion	Died within 24hours after delivery	Died 1–42 daysafter delivery	Total
HIV	23 (31.1)	28 (37.8)	2 (2.7)	21 (28.4)	74
Malaria	14 (63.6)	3 (13.6)	0 (0.0)	5 (22.7)	22
TB	9 (56.3)	2 (12.5)	1 (6.3)	4 (25.0)	16
Anemia	6 (40.0)	2 (13.3)	3 (20.0)	4 (26.7)	15
Other Infections	6 (46.2)	2 (15.4)	0 (0.0)	5 (38.5)	13
Other [non-infectious] causes^a^	8 (53.3)	2 (13.3)	1 (6.7)	4 (26.7)	15
Undetermined	4 (44.4)	1 (11.1)	1 (11.1)	3 (33.3)	9
Total	70 (42.7)	40 (24.4)	8 (4.9)	46 (28.0)	164

a Other [all other non-infectious causes] (15) = liver (1), CVD (2), injuries (3), cancers (2), gastro (2), kidney (1), lung (1), CNS (2), other (1).

### Circumstances Surrounding Pregnancy-related Deaths

#### Deaths during pregnancy

Of 92 PR deaths that occurred during pregnancy, the median gestational age at time of death was 5.5 months (SD 2.3), with 26% of women dying in the first, 38% in the second, and 36% in the third trimester. Nearly two-thirds (61%) of deaths in pregnant women reportedly had a high fever before death, and of these, 29%, 45%, and 26% occurred in the first, second and third trimester, respectively. Vaginal bleeding was reported in 18 (23%) of 77 deaths during pregnancy where information was recorded and of these, 30%, 30%, and 40% occurred in the first, second and third trimester, respectively. Seizures were reported in 7 (9%) of 76 deaths during pregnancy where information was recorded; of these 17%, 56%, and 33% occurred in the first, second and third trimester, respectively. Of 77 pregnancy-related deaths with a record on past complications, 11 (14%) had a history of a previous complicated delivery. A higher proportion of these women died later in pregnancy (χ^2^ linear trend = 6.7, p = 0.01), with 10% of these deaths occurring in the first, 30% in the second and 60% in the third trimester. Subjectively described “high fever” before death during pregnancy declined from 94% of deaths in 2003, to 30% by 2007 (χ^2^ linear trend = 11.0, p = 0.001). The new form in 2008 did not record this data. There was no difference in the frequency of reported high fever among pregnant women who reportedly died from an infectious disease, compared with other causes.

The reported symptom of “vaginal bleeding” during pregnancy (which was differentiated from peripartum hemorrhage) (n = 18) was associated with older age, with only four women below 30 years (p = 0.004) reporting this symptom; 29% of vaginal bleeding occurred in the first, 30% in the second, and 41% in the third trimester of pregnancy. A third of women who reported to have had a vaginal bleeding had a history of past delivery complications, compared with 9% of other pregnancy deaths (p = 0.01). Women with vaginal bleeding during pregnancy were more likely to die at or on route to hospital or a health facility than women who had no reported history of vaginal bleeding during pregnancy (p = 0.02), with half dying in the hospital.

Seven women who died during pregnancy had recorded seizures across all trimesters, with none in the 9^th^ month. Seizures occurred across ages and socio-economic groups, with half reported in 2006–2007. All but one case died at home. Eclampsia was defined as the cause of death by VA for 2/7 (29%). In the remaining women whose deaths were associated with seizures and who died during pregnancy, the cause of death defined by VA was TB (1 death), HIV (2 deaths), asthma (1 death) and dysentery (1 death).

#### Women who died following a miscarriage/abortion

**Figure 3:**
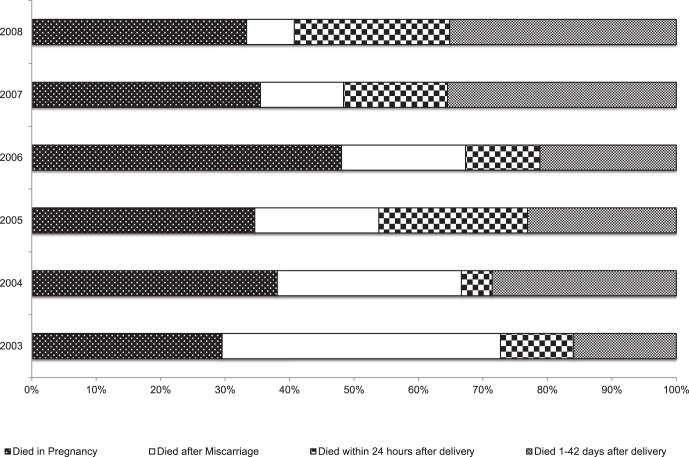
Proportion of pregnancy-related deaths by phase of pregnancy and year of death.* *Note - prior to 2008, VA questionnaire asked for all miscarriages/abortions (spontaneous or induced); as of 2008, the WHO VA questionnaire was adopted which only asked for induced abortions.

54 deaths were associated with miscarriage; reported deaths associated with miscarriages dropped significantly over time (χ^2^ linear trend = 5.0, p = 0.03), with over half (57%) having been reported in 2003/2004 and <15% in 2007/2008. Thus, the contribution of miscarriages/abortions to PR deaths fell from 43% in 2003 to 7% in 2008 (Figure 3). Of note, a change in the VA questionnaire in 2008 limited the VA recording of miscarriages when they were induced. Although miscarriage/abortion associated deaths occurred across all age groups, the highest proportion (40%) of miscarriages/abortions was among women 25–29 years. The mean number of reported days until death occurred following miscarriage/abortion was 16 days (SD 27; range 1–90 days), with 70% of deaths occurring within one week of the miscarriage/abortion. HIV/AIDS was attributed as the direct cause of death in 52% of all miscarriages/abortion, with a further 6% and 4% associated with malaria and TB, respectively. Reportedly, 10% of miscarriages/abortions had been induced; among these, none were married at the time of death, compared with 60% of women being married who had a miscarriage/abortion that was non-induced (p = 0.011). Over two-thirds (69%) of deaths associated with miscarriage/abortion occurred at home.

#### Postpartum deaths (up to 42 days post-delivery)

Of 103 deaths in the postpartum period, 37% died within 24 hours, 68% died by the end of the first week, and 97% within one month, with a median of 9 days (range 40) between delivery and death. The majority of postpartum deaths occurred in younger women, and only 20% in women over 35 years. Nearly half of postpartum deaths (45%) were among women in the two lowest SES quintiles. Half (52%) of all post-delivery deaths were classified as maternal deaths, 23% were attributed to HIV/AIDS, and the remainder attributed to nutritional, TB, other infections, malaria, and other causes. Most deaths that occurred within 24 hours of delivery were attributed to direct obstetric causes compared to deaths that occurred at least 24 hours after delivery (82% vs. 33%, p<0.001). A higher proportion of postpartum deaths that occurred at least 24 hours after delivery were HIV/AIDS related, compared to HIV/AIDS related deaths in the first 24 hours after delivery (34% versus 5%, p = 0.001).

The majority (89%) of women who died postpartum had delivered vaginally, 10% were by Caesarian-section, and in 1% forceps were used. VA-reported labor lasting longer than 24 hours occurred in 38% of cases and 28% of all labors were considered to be prolonged. Of postpartum deaths with information recorded on events in the peripartum period, 42% of the deaths were reported to have had an obstructed labor, 20% bled heavily before delivery, and 33% bled heavily after delivery.

Of 77 maternal death reports with known place of delivery, nearly two-thirds (61%) of women delivered at home. Forty-two percent of home deliveries and 89% of hospital deliveries were in women from a higher SES (MCA 3–5). 82% of deliveries conducted by health professionals were among women in the higher SES group, while 68% of those delivered by a TBA were in the aggregated lower SES group (p = 0.02; MCA 1–2). The proportion of post-partum deaths amongst all PR deaths increased between 2003 and 2008, both among immediate post-partum, and for those occurring from 1–42 days (Figure 3).

Of 37 deaths in the immediate (24 hours) postpartum period, 25 had delivery information; 48% delivered at home, and 44% delivered in a hospital or health facility; 40% of women dying post-partum were delivered by a health professional, 32% by a TBA, 16% by a relative, and 12% by themselves. Forty-nine out of 66 deaths that occurred 1–42 days in the postpartum period had information available on who performed the delivery; less than a third (29%) of deliveries were performed by a health professional in a hospital or health facility.

Nearly all (91%) of the 22 deaths due to PPH occurred within 24 hours of delivery; over a third (36%) of whom were women over 35 years. Of the 22 PPH deaths, 14 (64%) died at home; all within 24 hours. Among 13/14 home deliveries who died of PPH, where details were given, eight recorded who helped at the delivery: three had a TBA, in three, only relatives were present, and two delivered themselves. Thus, among known PPH cases delivering at home, 63% of the deliveries were conducted by the patient or relatives.

#### Birth outcomes among women who died post-partum

Among 76 postpartum deaths with birth outcome recorded, 79% had babies born alive. VA documented 39/57 (68%) of babies had died by the time of follow up; 32% died within 7 days, and 36% died after 7 days. By HDSS follow-up, 21% of babies were healthy and 11% were not thriving. Significantly more (85%) babies were born alive among mothers surviving the first day, compared with 64% of those whose mother died in the first 24 hours post-partum (p = 0.04). A higher proportion of babies were born alive outside of health facilities compared with in-facilities (90% versus 58%, p = 0.02).

#### Care seeking before death among women who died of pregnancy-related and non-pregnancy-related causes

Prior to death, 89% of women classified as PR deaths sought some type of care outside of the home, compared with 98% of non-PR deaths (p<0.001). While 73% and 72% of women with PR and non-PR related causes of death, respectively, sought some form of hospital care before death, a review of other types of care sought illustrates significant differences in sources of care among PR and non-PR deaths: religious leaders (30% versus 39%, p = 0.004), pharmacy or drug seller (52% versus 71%, p<0.001), and health facilities (47% versus 58%, p = 0.002). While a higher proportion of women with a PR death sought help from a TBA (18%), compared with deaths that were non-PR (15%) this was not statistically significant (p = 0.19). When separating out women who died within or after 24 hours of delivery, 54% of those dying within 24 hours had sought care from a hospital, physician or health facility, compared with 89% of women who died later post-partum (p<0.001).

SES was not associated with health seeking in general; however differences were evident by type of care sought. Thus, while 25% overall visited a traditional healer, the proportion increased as SES decreased, with 31% visiting traditional healers in the lowest MCA quintile compared with 16% in the highest (χ^2^ linear trend 4.2, p = 0.04). Similarly, 34% of the poorest sought care from TBAs compared with 12% in the highest MCA quintile (χ^2^ linear trend = 4.5, p = 0.034). The reverse was evident for hospital care, with 90% of women in the highest MCA quintile seeking hospital care compared with 60% in the lowest (χ^2^ linear trend = 11.4, p = 0.001).

## Discussion

During the six year period between 2003 and 2008, we estimated a pregnancy-related mortality ratio of 740 (95% CI 651–838) per 100,000 live births within the KEMRI/CDC Health and Demographic Surveillance System in Nyanza Province, western Kenya with no evidence of reduction over time. If we remove all incidental or accidental causes of death as ascribed by VA, as well as deaths due to undetermined causes, the resulting maternal mortality ratio is 669 (95% CI 584–762) per 100,000 live births. This estimate is higher than the national estimate of 488 (95% CI: 333–643) per 100,000 live births reported by the Kenya Demographic and Health Survey in 2008/2009 for approximately the same time period, and far above the national target of 147 per 100,000 by 2012 [22]. The lifetime risk of dying from pregnancy-related causes in the HDSS was 1 in 26, in contrast to over 1 in 7300 for women from developed countries, underscoring a very large inequity in maternal mortality [6].

Nearly one-third of deaths were due to direct obstetric causes. Postpartum hemorrhage was the leading obstetric cause of death, which is likely a direct reflection of the lack of access to timely and competent hospital care. Most women who died of PPH delivered at home, where essential skills and interventions (misoprostol and safe blood) are not available, and timely transfer to health facilities during a time of obstetric emergency is difficult. The majority of peripheral health facilities in the HDSS also lack misoprostol, safe blood, and appropriate septic techniques to manage the leading causes of obstetric complications in this area (Sheila Ogwang, personal communication). Complications associated with abortion/miscarriage were also a common direct cause of PR deaths, and all of these deaths were in unmarried women. Data from ongoing studies in the HDSS suggest that 80% of pregnancies are unintended [23]; 75% of women of childbearing age in this area do not have access to family planning (HDSS, unpublished data), compared to the national [3] and SSA [24] estimate of 25%. This gap can largely be attributed to inadequate service provision, unreliable access to family planning commodities and limited resource allocation nationally. Furthermore, unmarried females may have difficulty accessing ‘family planning’ because contraceptives may be considered only appropriate for married women [25]. In this context, improved access to family planning commodities and implementation of interventions to reduce the risk of death posed by unintended pregnancies and unsafe abortions [7] should be considered.

Among pregnancy-related deaths recorded in this study, only 33% were delivered by a skilled birth attendant (health worker with midwifery skills). Nationally, the proportion of births managed by health professionals and the proportion delivered in a health facility are 44% and 43%, respectively [3]. Skilled birth attendance in the HDSS has improved from 17% in 2002 [26] to 39% in 2010 (KEMRI/CDC, unpublished data), most likely as a result of policy changes and maternal health initiatives to improve access to and removal of barriers for the use of skilled birth attendants. This improvement in skilled birth attendants, however, fell short of the global target of 85% skilled birth attendants for 2010 [27].

There was no difference in seeking care from a TBA between PR and non-PR deaths, which implies that in this community, TBAs may be providing care to both pregnant and non-pregnant/non-parturient women. Furthermore, women of lower socioeconomic status were more likely to deliver at home and less likely to seek care from a health facility any time before their death. A program that encourages TBAs to accompany women in labor to the health facility from their community, and allows TBAs to provide emotional support during labor and delivery may increase skilled birth attendance and improve maternal and perinatal outcomes in rural Kenya [28], [29], while still involving these important community members. Furthermore, closing the gap on availability of and access to facilities offering emergency obstetric care (EmOC), along with targeted health education and support during pregnancy, improvements in the transport and communication referral network, and provision of free high quality maternity services should also help with realization of the MDG5 goals in this area. These changes should ideally be accompanied by a systematic evaluation of the quality of care provided to women presenting to health facilities for delivery. Efforts to improve skilled birth attendance in the HDSS have been accelerated since 2010. Only 5 (14%) of the 36 health facilities in the HDSS provided some level of EmOC in 2002, and after nearly a decade of multiple efforts to improve skilled birth attendance, 2 (6%) offered comprehensive EmOC, one quarter (9 facilities) offered the full spectrum of basic EmOC services, and 21 offered some level of basic EmOC (missing one or more of the key services); while 4 did not offer any obstetric care services by the end of 2011 (Sheila Ogwang, personal communication).

Almost one-third of the pregnancy-related deaths that occurred in a health facility or hospital had delivered at home, providing an example of the “Three Phases of Delay Model” [30]: delay in recognition of illness, delay in seeking and accessing care, and delay in the provision of care once at a health facility. Family influence, cost and poor healthcare staff attitude have been cited as reasons why women do not seek facility-based delivery services in this area [31]. Poor road infrastructure and lack of functional ambulatory services further hinders referrals and access to EmOC. Our data also suggest that women who are successful in reaching the health facility for a delivery should be kept under observation for at least 24 hours following the delivery, as one-third of the pregnancy-related deaths from a directly attributable cause occur within that period.

According to VA findings, nearly two-thirds of deaths were due to indirect, non-obstetric causes. This contrasts to a recent WHO analysis on maternal deaths in Africa which suggests that the majority of deaths are due to direct obstetric complications around the time of childbirth [5]. Our study is in agreement with an autopsy study of maternal mortality at a tertiary health facility in Mozambique which highlights the importance of infectious causes of maternal death [32]. In our study, HIV/AIDS, malaria, TB and anemia accounted for 30%, 9%, 6% and 6% of all pregnancy-related deaths, respectively [33], [34], [35]. TB in association with HIV/AIDS is increasingly recognized as a major contributor to maternal mortality in SSA [36]. Our findings suggest an opportunity to implement a streamlined program of integrated point-of-care detection and appropriate management of these major pregnancy-related illnesses in a setting where 92% of pregnant women attend at least one antenatal care visit [3]. It also highlights the need to improve access to and increase uptake of prevention and treatment measures against these infections.

According to the VA, malaria was a significant ascribed cause of pregnancy-related death (65 per 100,000 live births). These data add to the scarcity of literature documenting the impact of malaria on maternal mortality in Africa [37]. It has commonly been assumed that malaria in pregnancy is associated with death predominantly in areas of unstable or low malaria transmission [32], [38]. However, an earlier review suggests that the overall percentage of maternal deaths attributed to malaria from direct and indirect causes in low transmission areas outside of Africa (0·6–12·5%) is not substantially different from the estimate derived for high transmission areas (0·5–23·0%) [39]; our data support this finding. Within the study area in past years, interventions have been conducted to reduce the harmful effects of malaria in pregnancy, specifically with insecticide-treated bednet distribution for pregnant women [40], intermittent presumptive treatment with sulfadoxine-pyrimethamine [41], [42], and training of healthcare workers to provide focused antenatal care [17]. However, assessments of outcomes from these interventions have generally focused on the impact on low birth-weight and neonatal mortality, but not on reduction in pregnancy-related mortality. More data are required in determining the effect of these and other antenatal care interventions on maternal survival.

Our evaluation was subject to several limitations: 1) In the absence of linkage to antenatal care data, we were unable to fully ascertain the health status of pregnant women before they died; 2) The small number of pregnancy-related deaths resulted in wide confidence intervals and likely contributed to substantial fluctuations in annual estimates of pregnancy-related mortality. Future analyses over longer time periods should allow for more precise estimates as well as evaluation of the impact of current interventions on maternal mortality; 3) Since national estimates are not disaggregated by province or even rural/urban settings, we are unable to make any comparisons at that level; however, our data contribute much needed sub-national mortality information to prioritize interventions [43]; 4) Of all deaths, over 90% are followed up with VA in the community, the remaining not being followed due to migration, absence of an adult or next of kin at the time of household visit. VA is expected to underestimate pregnancy-related deaths by about 15% [44] due to recall bias and the absence of diagnostics. Furthermore, questioning of symptomatology around obstetrical complications is particularly difficult, and likely misses a number of clinical signs not witnessed by relatives or other respondents. This potentially under-estimates some symptomatology around PR deaths, limits our interpretation of clinical events, and prevents chronological evaluation of events leading up to the time of death. In most SSA countries, including Kenya, pregnancies are not declared early [43] which further reduces the likelihood of capturing all pregnancy-related deaths through VA. That the majority of miscarriages/abortions and post-partum deaths were classified under other causes raises our suspicion that mortality from this cause is under-reported. In addition, cultural factors appear to act as a strong barrier against disclosure of miscarriage in the HDSS community (Stephanie Dellicour, personal communication); 5) Changes in the verbal autopsy questionnaire on miscarriage in 2008 may have also reduced ascertainment in that year, thus this may have exaggerated the proportionate fall in miscarriage, relative to other indirect causes. Further, legalities surrounding induced abortion likely also influenced accurate reporting of such events; 6) We recognize within-death comparison reflects behaviors of selected sub-populations, but for many variables no comparative data were available among healthy deliveries; 7) We did not use the WHO ICD definition of maternal mortality but instead chose to use its definition of pregnancy-related mortality which includes deaths due to incidental or accidental causes. While there is very limited literature on external causes of death during pregnancy [45], [46], [47], and the relationship between pregnancy and such external causes is likely to be complex and context-specific, we believe that incidental deaths may, at least in part, be pregnancy-related in resource-poor settings such as western Kenya where scenarios like the poor care of a pregnant woman diagnosed with cancer or domestic violence during pregnancy cannot be underestimated; and 8) Ascertainment of changing mortality trends depends on a robust ongoing surveillance system. The HDSS has been in existence since a large insecticide-treated bednet trial, and improved with membership into INDEPTH with standardization of forms to enhance capture of signs and symptoms informing diagnosis [10]. Since 2003, capture of deaths has been standardized, yet cyclical variations by year are evident; the lower than expected mortality rate in 2005, followed by a higher rate in 2006 was also documented within other sub-populations in the HDSS; while no clear explanation for this occurrence is evident, it appears to be a population level phenomenon rather than maternal-specific. We recognize verbal autopsy has numerous limitations in its ability to define cause of death; new approaches to analyzing symptomatology with classification by WHO ICD-10 codes is underway, such as the use of a computerized algorithm [48], [49] which is hoped will strengthen future analyses. Despite these limitations, we believe these data represent unique and robust information from SSA and contribute to the literature about events surrounding pregnancy-related mortality.

### Conclusions

In a rural area of western Kenya, we observed very high pregnancy-related mortality, the leading causes of which were HIV/AIDS, malaria, postpartum hemorrhage, TB, anemia, miscarriage/abortion, and puerperal sepsis. Data from the HDSS, and VA identifying underlying causes, suggest most public health surveillance systems in areas of high disease burden may be underestimating certain causes of PR deaths. Over two-thirds of the pregnancy-related deaths identified over the study period were preventable. Our data also attest to the limited care seeking currently adopted by women during pregnancy, with substantial numbers seeking care from traditional practitioners. There is potential to substantially reduce pregnancy-related mortality in rural Kenya, and indeed in SSA, by targeting endemic infectious diseases prior to and during pregnancy, as well as ensuring all pregnant women have access to good quality antenatal care, skilled care during delivery, emergency obstetric care as well as post-natal care. In the past few years, several development partners and U.S. government organizations across SSA have allocated limited resources towards maternal health programs. Our findings show that these efforts need to be targeted towards improving pregnancy-related and maternal health care both at home (through community health advocacy and education) and at health facilities (through improved healthcare worker training, supervision, and guidelines in provision of antenatal and obstetric care). It is time to implement and measure the impact of a targeted, evidence-based approach to reducing pregnancy-related mortality in Kenya. As one practicing gynecologist and head of a district hospital in rural western Kenya said, *“No woman should die while giving forth life*”.
